# Retraction: Maize *opaque10* Encodes a Cereal-Specific Protein That Is Essential for the Proper Distribution of Zeins in Endosperm Protein Bodies

**DOI:** 10.1371/journal.pgen.1010501

**Published:** 2022-11-16

**Authors:** The PLOS Genetics Editors

After publication of this article [1], concerns were raised about several of the figures as well as inconsistencies between the Materials and Methods and the Results. Specifically, for the figures:

- The Anti-BIP 21DAP WT and *o10* bands in Fig 4G appear similar to the anti-tubulin panel in S2 Fig F. The corresponding author stated that the western blots in S2 Fig F were assembled in error and provided an updated S2 Fig F where both western blot panels have been replaced with replicate experiments from the time of the original experiments.
- The right three lanes in the anti-*o10*-C panel in Fig 5A appear similar to the right three lanes in an anti-*o10* panel in [2] when reflected vertically with altered exposure and contrast. The corresponding author acknowledged the images are likely the same and stated that they do not know which image is correct.
- Fig 5B results do not appear to be consistent with the *o10* localisation diagrams in Fig 6B which appear to represent ten pictures of PBs for the same assay. The corresponding author stated that Fig 6B is a diagram that summarises the general distribution patterns of *o10* and zeins, but the dots do not strictly correspond to data from any given electron microscope image.
- In S4 Fig B, lanes 2 and 3 in the anti-*o10* panel appear similar to an anti-tubulin panel in Fig 3 16B of [2] when rotated 180°. The corresponding author stated the images are likely the same but that S4 Fig B in [1] is correct.

The corresponding author stated that the original data underlying the results in Figs 2B, 2D-E, 4E-H, 5A-B, 5D, 6A-B, 7A-B, S1 D-F, S2 A, S2 C, S2 E-F and S4 A-B are no longer available.

Several reporting errors were also identified in the Methods and Results.

Due to unresolved figure concerns, the extent of reporting errors, and the unavailability of a substantial amount of the underlying data for this study, the *PLOS Genetics* Editors retract this article [1].

RS did not agree with retraction. DY, WQ, XL, QY, SY, HL, GaW and GuW did not respond or could not be reached.

RS stands by the article’s findings and apologizes for the issues with the published article.
